# Exploring CT Texture Parameters as Predictive and Response Imaging Biomarkers of Survival in Patients With Metastatic Melanoma Treated With PD-1 Inhibitor Nivolumab: A Pilot Study Using a Delta-Radiomics Approach

**DOI:** 10.3389/fonc.2021.704607

**Published:** 2021-10-07

**Authors:** Antonino Guerrisi, Michelangelo Russillo, Emiliano Loi, Balaji Ganeshan, Sara Ungania, Flora Desiderio, Vicente Bruzzaniti, Italia Falcone, Davide Renna, Virginia Ferraresi, Mauro Caterino, Francesco Maria Solivetti, Francesco Cognetti, Aldo Morrone

**Affiliations:** ^1^ Radiology and Diagnostic Imaging Unit, Department of Clinical and Dermatological Research, San Gallicano Dermatological Institute IRCCS, Rome, Italy; ^2^ Medical Oncology Unit 1, Department of Clinical and Cancer Research IRCCS Regina Elena National Cancer Institute, Rome, Italy; ^3^ Medical Physics and Expert Systems Laboratory, 3 Department of Research and Advanced Technologies, Istituti Fisioterapici Ospitalieri - IRCCS Regina Elena National Cancer Institute, Rome, Italy; ^4^ Institute of Nuclear Medicine, Imaging Department, University College Hospital, London, United Kingdom; ^5^ Medical Oncology 1, IRCCS-Regina Elena National Cancer Institute, Rome, Italy; ^6^ Scientific Director, San Gallicano Dermatological Institute IRCCS, Rome, Italy

**Keywords:** melanoma, immunotherapy, x-ray computed tomography, biomarker, precision medicine, image analysis, radiomics

## Abstract

In the era of artificial intelligence and precision medicine, the use of quantitative imaging methodological approaches could improve the cancer patient’s therapeutic approaches. Specifically, our pilot study aims to explore whether CT texture features on both baseline and first post-treatment contrast-enhanced CT may act as a predictor of overall survival (OS) and progression-free survival (PFS) in metastatic melanoma (MM) patients treated with the PD-1 inhibitor Nivolumab. Ninety-four lesions from 32 patients treated with Nivolumab were analyzed. Manual segmentation was performed using a free-hand polygon approach by drawing a region of interest (ROI) around each target lesion (up to five lesions were selected per patient according to RECIST 1.1). Filtration-histogram-based texture analysis was employed using a commercially available research software called TexRAD (Feedback Medical Ltd, London, UK; https://fbkmed.com/texrad-landing-2/) Percentage changes in texture features were calculated to perform delta-radiomics analysis. Texture feature kurtosis at fine and medium filter scale predicted OS and PFS. A higher kurtosis is correlated with good prognosis; kurtosis values greater than 1.11 for SSF = 2 and 1.20 for SSF = 3 were indicators of higher OS (fine texture: 192 HR = 0.56, 95% CI = 0.32–0.96, *p* = 0.03; medium texture: HR = 0.54, 95% CI = 0.29–0.99, *p* = 0.04) and PFS (fine texture: HR = 0.53, 95% CI = 0.29–0.95, *p* = 0.03; medium texture: HR = 0.49, 209 95% CI = 0.25–0.96, *p* = 0.03). In delta-radiomics analysis, the entropy percentage variation correlated with OS and PFS. Increasing entropy indicates a worse outcome. An entropy variation greater than 5% was an indicator of bad prognosis. CT delta-texture analysis quantified as entropy predicted OS and PFS. Baseline CT texture quantified as kurtosis also predicted survival baseline. Further studies with larger cohorts are mandatory to confirm these promising exploratory results.

## Introduction

Immunotherapy is changing the landscape of oncology (1, 2). In particular, immune checkpoint inhibitors such as programmed cell death protein 1 (PD-1) inhibitors are demonstrating an increased overall survival and progression-free survival (OS and PFS) in patients with metastatic melanoma (MM) (2–5). However, some patients benefit less than others and many factors are involved in the varied response rates (6). Intra/inter-tumor heterogeneity (ITH) may represent one of the reasons why some patients with MM do not gain real benefits from immunotherapy (7).

Early identification of non-responding patients avoids potential unwanted side effects and reduces the economic burden associated with unnecessary treatments on healthcare providers (8). The development of robust biomarkers for immunotherapy response represents an ongoing important challenge and focus area for research and development (9–11). Although many predictive markers for immunotherapy response have been investigated in MM, validated reliable biomarkers have not yet been identified (12).

Nowadays, imaging criteria (i.e., RECIST 1.1) based on computed tomography (CT) remains the gold standard for evaluating treatment response in clinical trials (13). CT remains indispensable for diagnosis and follow-up as it is reproducible, standardized, and suitable for extracting qualitative–quantitative data (14). However, imaging patterns of the immune mechanism and its associated/atypical response in some patients significantly differ from those seen with the use of more common cytotoxic agents (15). Indeed, treatment response after immunotherapy can be associated with pseudo-progression or hyper-progression (15). To overcome RECIST 1.1 limits, other imaging criteria (i.e., irRECIST, irRC, and iRECIST) have been proposed in clinical trials (16, 17). However, since available evidence for these criteria is still limited in melanoma and may not fully capture all patterns of clinical responses, caution is recommended in the use of these criteria in routine clinical practices (18). Thus, there is an urgent need to look for predictive biomarkers of immunotherapy response in patients with MM in order to provide robust and objective clinical end points.

Radiomics is a promising field of research that uses quantitative imaging methodological approaches that could support the oncologist in treatment decisions (19, 20). An image-based radiomics approach can analyze quantitative information from the whole tumor volume and the multiple lesions located in different anatomical sites within a single examination (21). CT texture analysis (CTTA) provides a panel of quantitative parameters reflecting intra/inter-tumor heterogeneity associated with a high-risk phenotype (22, 23). The delta-radiomics approach allows the evaluation of baseline and post-therapy changes in texture features within target lesions, in order to determine temporal changes in tumor heterogeneity (24).

The purpose of this study was to explore features extracted on baseline CT and post-treatment (delta-radiomics features) as predictors of OS and PFS in patients with MM treated with the PD-1 inhibitor Nivolumab, and its ability to act as a novel imaging biomarker for predicting survival.

## Materials and Methods

### Study Population

**Figure 1:**
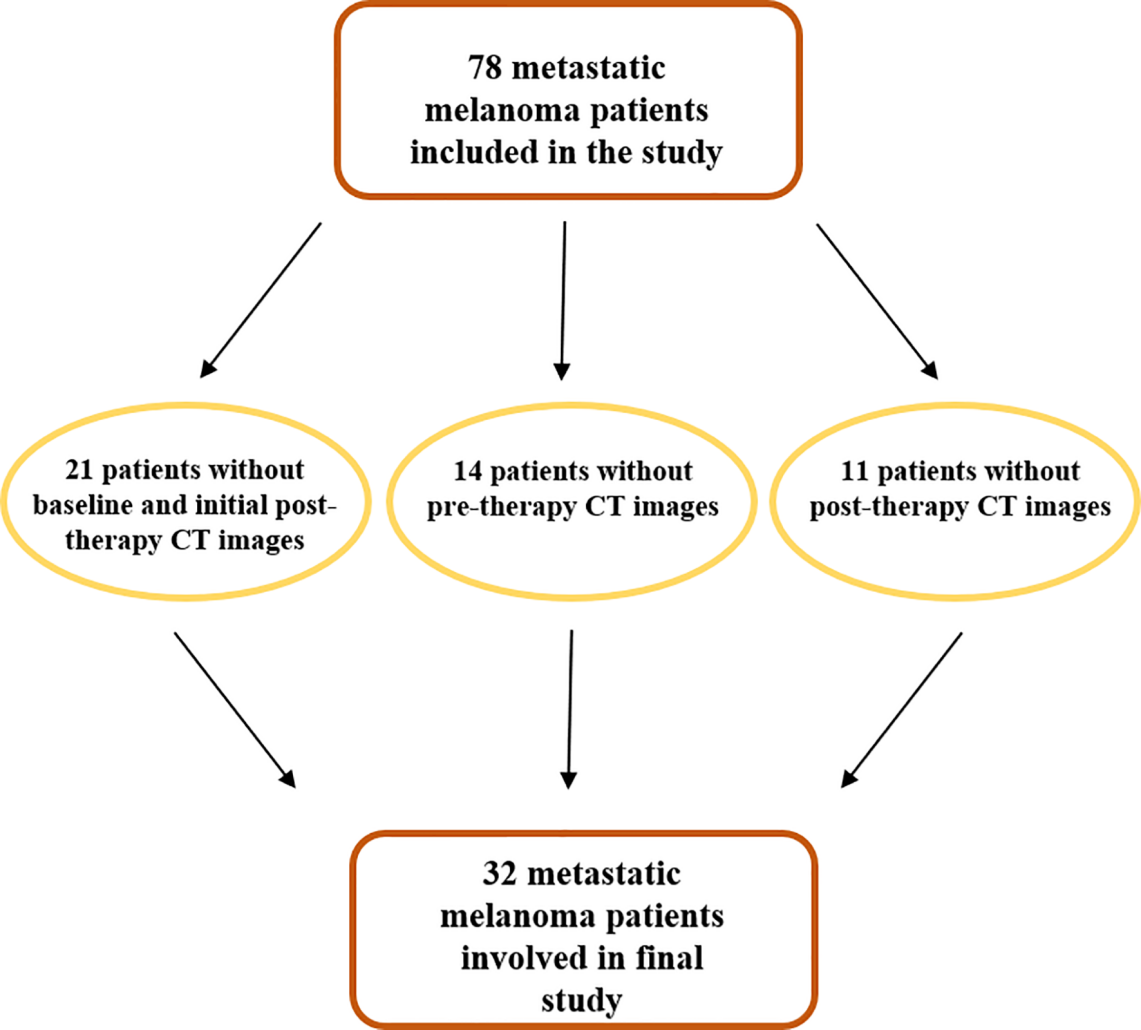
Schematic characteristics of patients enrolled in the study.

Institutional review board approval was obtained for this retrospective study with a waiver of informed consent. At first, 78 MM patients treated with anti-PD-1 therapy, from January 2015 to February 2019, were included in the study. Patient data were extracted from our institutional oncologic digital database and RIS/PACS system (Centricity RIS/PACS, GE-Healthcare). Patients were included in the study only if they had both a contrast-enhanced CT and LDH measurements obtained at baseline/pre-therapy (within 1 month before the start of therapy) and initial post-therapy (about 3 months from the start of therapy). Crucial inclusion criteria of patients in this study required that contrast CT had been performed on the same scanner and with the same institutional protocol for the whole body. Schematic evolution of study population is represented in **Figure 1**.

### Survival End Points

OS and PFS were chosen as end points. OS was defined as the time between the beginning of PD-1 inhibitor therapy and the death of patients. PFS was defined as the time between the beginning of PD-1 inhibitor therapy and the evidence of progressive disease (PD) at CT examination, according to RECIST 1.1. Patients, alive or without evidence of PD at the end of the follow-up, were censored to the last follow-up visit.

### Clinical Markers

Clinical variables considered for multivariate analysis were serum lactate dehydrogenase (LDH), the percentage change in LDH before and after therapy (PERC-LDH), and the number of metastatic sites involved (4, 25). All clinical variables were dichotomized to the following cutoff points based on the literature: LDH cutoff = 1.5 × upper limit of normal, PERC-LDH cutoff = 20%, and number of metastatic sites cutoff = 3 (11, 25).

### CT Examination

CT scans were acquired within a month prior to the start of therapy and, subsequently, every 3 months or in the presence of clinical signs suggesting PD. Images were acquired using a 128 MDCT scanner (Brilliance CT, Philips Healthcare), 64 × 0.625 (128) detector width (mm). The tube voltage was 100 kVp; the tube current product was determined using the automatic tube current modulation (ATCM) technique (Dose Right, Philips Healthcare), with 200 mAs as a reference parameter to minimize radiation exposure. Other scan parameters were as follows: pitch, 0.891; rotation time, 0.4 s; field of view (FOV), 350 mm; slice thickness, 2.5 mm; slice increment, 1.25 mm; matrix 512 × 512; pixel spacing, 0.98 mm × 0.98 mm. After an initial non-contrast scan, all patients received intravenous contrast (Ultravist^®^ 370; Schering AG and Iomeron^®^ 400; Bracco Imaging Italia s.r.l.) at a dose of 1.3 ml/kg and 1.5 ml/kg, respectively, and both at a rate of 3.0 ml/s *via* a 20-gauge catheter placed in the antecubital vein, followed by 40 ml of saline at the same rate. A multiphasic scan was initiated at 15, 55, and 150 s after CT attenuation of the aortic lumen at the level of the celiac trunk, reaching the trigger attenuation threshold of 150 HU, covering the whole body. Raw data of CT scans were reconstructed with a standard filtered back-projection algorithm in the DICOM format, according to institutional protocol.

### Region of Interest Segmentation and CT-Based Radiomic Texture Analysis

Manual segmentation was performed by drawing a region of interest (ROI) around each target lesion. Two radiologists with at least 10 years’ experience in oncologic and melanoma imaging, A.G. and F.M.F., evaluated target lesions, applied the RECIST 1.1 criteria, and drew the ROIs in consensus; successively, the same radiologists have evaluated therapy response (up to five lesions were selected per patient). We basically employed what is normally done in routine clinical practice when assessing response to treatment in these ontological patients to be as close and relevant to current practice. No bin width or size was employed, but each individual integer value in the unfiltered and filtered texture map was used (in other words integer binning) in the quantification of texture parameter-based histogram and statistical approach. Lesions with the largest diameter of less than 5 mm were excluded from the analysis. Indeed, smaller lesions will have fewer pixels/distribution of gray-level intensities whereby the statistics may not be optimum. Also, as the filtration-histogram-based texture analysis employed in this study uses a spatial scale filter (SSF) that extracts and enhances features of different sizes corresponding to the SSF value, to reduce the impact of lesion size on the quantification of texture metrics, we recommend that lesions should have a maximum diameter of at least 5 mm to provide a decent number of pixels (statistics)/gray-level intensity variation for extraction of texture features. We can still extract features within lesions <5 mm, but one may not be able to compute the different texture parameters at the higher SSF values. Each ROI was drawn on the slice through the largest diameter of the target lesion around the peripheral margin. Air, streak artifacts, and dense calcifications were excluded from the ROI. Texture feature extraction was performed on the baseline CT and the initial post-therapy contrast enhanced images (in DICOM format) acquired during venous phase. CTTA comprised a filtration-histogram technique. The filtration step, using a Laplacian of Gaussian band-pass filter (similar to a non-orthogonal wavelet), extracted and enhanced texture features of different sizes and intensity variation, corresponding to a SSF that varied from 2 to 6 mm: SSF = 2 corresponds to a fine texture scale, SSF = 3–5 corresponds to a medium texture scale and SSF = 6 corresponds to a coarse texture scale. Quantification of textures at each filter and for unfiltered data (SSF = 0) was undertaken using statistical and histogram-based metrics such as mean intensity (reflects average brightness), standard deviation (SD—reflects deviation from mean), entropy (reflects irregularity of pixel distribution), mean of positive pixels (MPP—only reflects average brightness of positive pixel values), skewness (reflects asymmetry of the histogram distribution), and kurtosis (reflects pointedness/sharpness of the histogram distribution). A detailed description of the above image filtration and quantification is described (26) and a computer modeling study characterized the meaning of filtration-histogram-based texture features in terms of image features and how they relate to the different components (object size, density, and number) of heterogeneity (23). For each patient, the average value of each texture feature from all lesions was calculated and used for the baseline analysis. Percentage changes in texture features on post-treatment from baseline scan were also calculated as part of the delta-radiomics analysis.

Percentage changes in the abovementioned features are indicated with the prefix “perc” and are calculated as follows:


$$\text{perc}-\text{Var}=100*\frac{var_{pre}-var_{post}}{var_{pre}}$$

**Figure 2:**
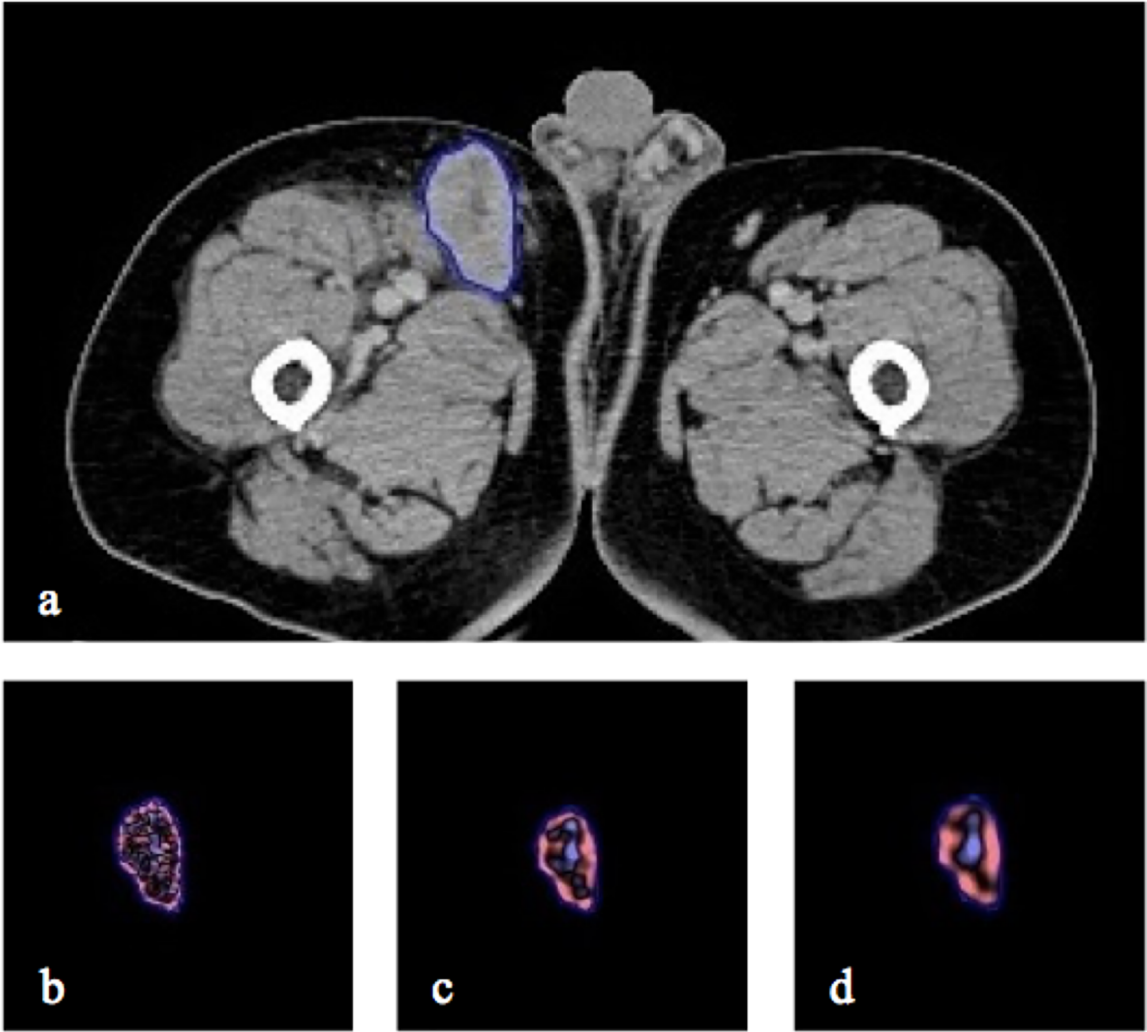
CT axial image with right inguinal lymph node segmented in 2D **(A)** and the resulting. Illustration of different image filtration as part of CTTA at **(B)** fine (SSF = 2mm), **(C)** medium (SSF = 4mm) and **(D)** coarse (SSF = 6mm) texture scales.

Where var_pre_ is the variable at baseline, var_post_ is the variable at initial post therapy and perc-Var is the percentage change. The whole process of ROI individual lesion segmentation and filtration histogram-based CTTA was undertaken using commercially available proprietary research software called TexRAD (Feedback Medical Ltd). **Figure 2** provides an illustration of the ROI segmentation and filtration-process as part of the CTTA.

### Statistical Analysis

The average values of each texture feature from all lesions were calculated for each patient on baseline CT and post-therapy CT and used for the statistical analysis. Average values of the texture metrics were calculated from all the lesions for each patient. This was done because we have the outcome (OS and PFS) per patient. The percentage change was computed for each lesion first and then averaged across all the lesions for each patient. The following steps were undertaken to find independent predictors of OS and PFS from the group of extracted features. Feature selection was performed using LASSO-Cox regression to identify the best predictors of OS and PFS. Indeed, LASSO improves the reliability of regression using a regularization parameter to reduce overfitting and selects optimal predictors. In fact, the LASSO method provides non-zero regression coefficients only for the best predictive features. In this way, it is possible to eliminate overfitting problems. Finally, among these, the feature with the coefficient whose numerical value was greater in absolute value was chosen. In this study, a 10-fold cross-validation was employed for every regression (27). A hazard ratio (HR) and 95% confidence interval (CI) was provided for the best univariate markers of OS and PFS. The best univariate texture predictors were included in the multivariate Cox analysis along with clinical markers, to assess the independence and/or interaction of the significant univariate texture markers in terms of predicting survival. For each feature that resulted as an independent predictor of survival in the multivariate Cox model, Kaplan–Meier (KM) survival curves/analysis differentiated between patients with good prognosis from poor prognosis, based on a median threshold to separate the two prognostic groups. Differences between survival curves were evaluated using a non-parametric log rank test. A two-tailed *p*-value of less than 0.05 indicated a significant difference. All statistical analyses were performed using R-package software (version 3.6.3; R Foundation for Statistical Computing).

## Results

### Patients Characteristics

Of the 78 patients analyzed for the study, only 32 (mean age 60 years; standard deviation 13.9) were really enrolled (see **Figure 2**). **Table 1** presents the demographics, clinical, and follow-up/survival information for all patients and sub-groups based on treatment response.

**Table 1:** Main demographic characteristics and relevant clinical data are reported in the table.

Variable	Total	Progression disease	Stable disease	Partial response
No. of patients	32	14	10	8
Age (years)	70 (29, 84)	67,5 (45, 82)	58 (29–79)	74 (54–84)
Sex (F, M)	9 F,23 M	6 F, 8 M	3 F, 7 M	8 M
No. of metastatic sites [≥3 (*N*), <3 (*N*)]	≥ (5), < (27)	≥ (3), < (11)	≥ (2), < (8)	≥ (0), < (8)
LDH pre therapy (UI/L)	389 (238, 827)	450 (313, 827)	331.9 (305–564)	367.5 (238–602)
LDH post therapy (UI/L)	419 (154, 803)	503 (202, 803)	390 (216–552)	287.5 (154–475)
LDH percentage Variation (%)	−3.73 (−50.16, 33.96)	9.28 (−49.6, 23.32)	−5.42 (−34.7–33.9)	−19.1 (−50.2–10.5)
ECOG	1 (0, 2)	1 (1, 2)	1 (0, 2)	0 (0, 1)
Hepatic lesions	Yes (9), No (23)	Yes (3), No (11)	Yes (3), No (7)	Yes (3), No (5)
Lung lesions	Yes (14), No (18)	Yes (9), No (5)	Yes (4), No (6)	Yes (5), No (13)
Mean target lesions size (mm)	30.4 (10, 140)	45.3 (10, 140)	38.7 (10, 88)	28.5 (10–40.3)
OS median/range (months)	31.6/2.73–66.8	20.6/2.73–48.6	38.6/3.5–56.6	54.6/29.3–66.8
PFS median/range (months)	15.1/2.5–52.7	5.3/2.6–24.3	23/2.5–50	46.1/13.2–52.6
Death	Yes (17), No (15)	Yes (12), No (2)	Yes (2), No (8)	Yes (3), No (5)

### Overall Survival Analysis

LASSO-Cox regression demonstrated baseline CT kurtosis at fine (SSF = 2) and medium (SSF = 3) texture scales predicting OS (fine texture: LASSO coefficient = −0.3; medium texture: LASSO coefficient = −0.35). A higher baseline kurtosis value was associated with good OS (fine texture: HR = 0.56, 95% CI = 0.32–0.96, *p* = 0.03; medium texture: HR = 0.54, 95% CI = 0.29–0.99, *p* = 0.04) (**Tables 2a, b**). LASSO-Cox regression also demonstrated delta-radiomics CT analysis, particularly the percentage change in entropy (Perc-ENTRO) at medium (SSF = 4) and coarse (SSF = 6) scales to predict OS (SSF = 4, LASSO coefficient = 0.02; SSF = 6, LASSO coefficient = 0.03). An increase in Perc-ENTRO was associated with poorer OS (SSF = 4, HR = 1.06, 95% CI = 1.01–1.11, *p* = 0.05; SSF = 6, HR = 1.06, 95% CI = 1.01–1.11, *p* = 0.01). A separate multivariate Cox regression analysis, which includes each significant univariate texture marker along with LDH and the number of metastatic sites, are presented in **Tables 2a–c**. Baseline CT kurtosis at fine (**Table 2a**) and medium (**Table 2b**) texture scales were predictors of OS, independent of LDH and the number of metastatic sites. Perc-ENTRO was a predictor at medium (SSF = 4) and coarse scales (SSF = 6) (**Tables 2c, d**).

**Table 2:** **(a–d)** Multivariate Cox regression analysis including individual significant univariate texture parameters (selected using LASSO) and clinical variables for predicting OS.

	Covariate	HR	95% CI	*p*-value
**a.**	Kurtosis (SSF = 2)	0.56	0.32–0.96	0.03
	LDH	0.29	0.07–1.12	0.09
	No. of metastatic sites	3.02	0.84–10.9	0.07
**b.**	Kurtosis (SSF = 3)	0.54	0.29–0.99	0.04
	LDH	0.33	0.08–1.21	0.10
	No. of metastatic sites	3.17	0.87–11.5	0.07
**c.**	Perc-ENTRO (SSF = 4)	1.06	1.01–1.11	0.05
	No. of met sites	4.02	1.14–14.9	0.04
	Perc-LDH	0.2	0.06–0.7	0.02
**d.**	Perc-ENTRO (SSF = 6)	1.06	1.01–1.11	0.01
	No. of met sites	4.25	1.14–15.8	0.03
	Perc-LDH	0.2	0.06–0.7	0.01

HR, hazard ratio; CI, confidence interval.

**Figure 3:**
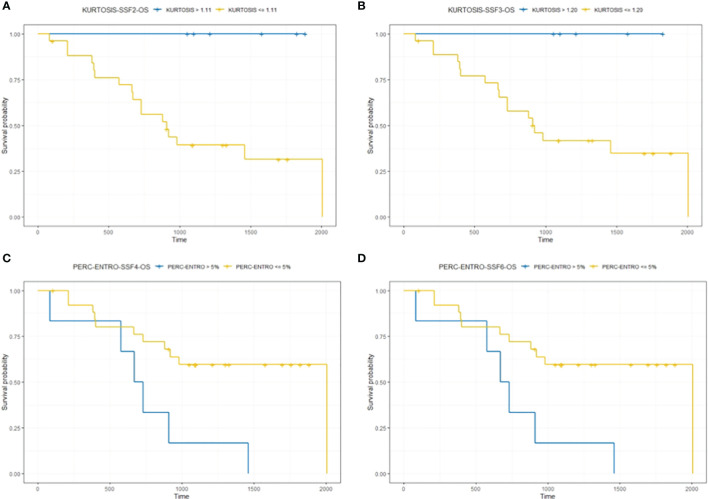
The figure compares survival curve for baseline CT texture parameter kurtosis at fine (SSF = 2) and medium (SSF = 3) texture scales and delta-analysis quantified Perc-ENTRO at medium (SSF = 4) and coarse (SSF = 6) scales for OS. Patients in the good prognostic group, as identified by baseline kurtosis are fine (SSF = 2) and medium (SSF = 3) texture scales, had an improved median survival of around 17 **(A)** and 10 months **(B)** respectively, compared to the poor prognostic group. Furthermore, the good prognostic group, which was defined using Kurtosis, demonstrated zero mortality. Patients in the good prognostic group, as identified by Perc-ENTRO at medium and coarse scales, improved median survival by around 11 months **(C, D)** compared to the poor prognostic group.

A Kaplan–Meier survival analysis for significant texture predictors of OS, based on their respective median cutoff, is presented in **Table 3**; Kaplan Meier survival curves are presented in **Figures 3A–D**.

**Table 3:** Kaplan–Meier analysis for significant texture predictors of OS.

Features	Cutoff	Median OS (months) for patients above the cutoff	Median OS (months) for patients below/equal to the cutoff	*p*-value
Kurtosis (SSF = 2)	1.11	46.6	29.8	0.01
Kurtosis (SSF = 3)	1.20	40.5	30.3	0.02
*Perc*-ENTRO (SSF = 4)	5	35.7	23.3	0.008
*Perc-*ENTRO (SSF = 6)	5	35.7	23.3	0.0005

### Progression-Free Survival Analysis

LASSO-Cox regression demonstrated baseline CT kurtosis at fine (SSF = 2) and medium (SSF = 3) texture scales predicting PFS (fine texture: LASSO coefficient = −0.35; medium texture: LASSO coefficient = −0.28). **Tables 4a, b** show that a higher baseline kurtosis value was associated with good PFS (fine texture: HR = 0.53, 95% CI = 0.29–0.95, *p* = 0.03; medium texture: HR = 0.49, 95% CI = 0.25–0.96, *p* = 0.03). LASSO-Cox regression also demonstrated delta-radiomics CT analysis, particularly Perc-ENTRO at medium (SSF = 4) and coarse (SSF = 6) scales to predict PFS (LASSO coefficient = 0.05 for SSF = 4, 0.04 for SSF = 5 and 0.04 for SSF = 6). An increase in Perc-ENTRO was associated with poorer PFS, for example, SSF = 4 (HR = 1.07, 95% CI = 1.01–1.13, *p* = 0.009). A separate multivariate Cox regression analysis, including each significant univariate texture marker along with LDH and a number of metastatic sites, is presented in **Tables 4a–c**. Baseline CT kurtosis at fine (**Table 4a**) and medium (**Table 4b**) texture scales were predictors of PFS, independent of LDH and number of metastatic sites. Perc-ENTRO and number of metastatic sites were independent predictors of PFS (**Tables 4c, d**).

**Table 4:** **(a–d)** Multivariate Cox regression analysis including individual significant univariate texture parameters (selected using LASSO) and clinical variables for predicting PFS.

	Covariate	HR	CI	*p*-value
**a.**	Kurtosis (SSF = 2)	0.53	0.29–0.95	0.03
	LDH	0.27	0.06–1.26	0.09
	No. of metastatic sites	2.59	0.75–8.9	0.13
**b.**	Kurtosis (SSF = 3)	0.49	0.25–0.96	0.03
	LDH	0.30	0.06–1.4	0.12
	No. of metastatic sites	2.62	0.76–8.99	0.13
**c.**	Perc-ENTRO (SSF = 4)	1.07	1.01–1.13	0.009
	No. of met sites	3.98	1.14–13.6	0.03
	Perc-LDH	0.28	0.07–1.1	0.07
**d.**	Perc-ENTRO (SSF = 6)	1.07	1.02–1.13	0.005
	No. of met sites	4.10	1.19–14.1	0.02
	Perc-LDH	0.27	0.07–1.1	0.06

HR, hazard ratio; CI, confidence interval.

**Figure 4:**
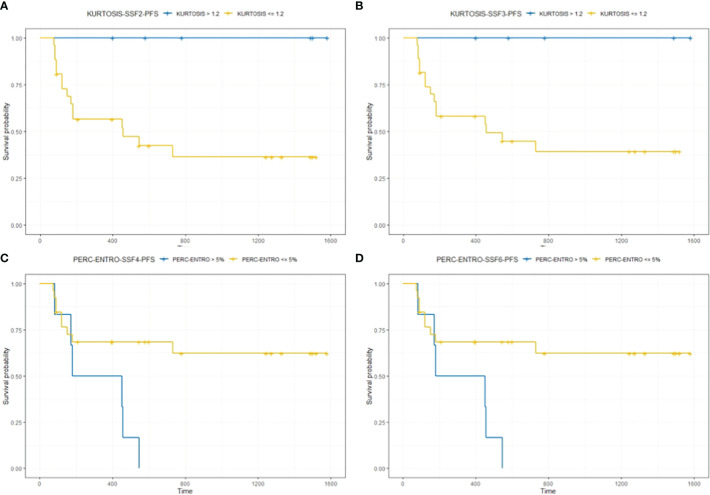
The figure compares survival curves for baseline CT texture parameter kurtosis at fine (SSF = 2) and medium (SSF = 3) texture scales, and delta-analysis quantified Perc-ENTRO at medium and coarse scales for PFS. Patients in the good prognostic group, as identified by baseline kurtosis at fine (SSF = 2) and medium (SSF = 3) texture scales, had an improved median PFS of around 28 **(A)** and 13 months **(B)** respectively, compared to the respective poor prognostic group. Furthermore, the good prognostic group defined using Kurtosis demonstrated zero progression. Patients in the good prognostic group, as identified by Perc-ENTRO, had an improved median PFS of around 8 months **(C, D)** compared to the poor prognostic group.

Kaplan–Meier survival analysis for significant texture predictors of PFS based on their respective median cutoff are presented in **Table 5**; Kaplan–Meier survival curves are presented in **Figures 4A–D**.

**Table 5:** Kaplan–Meier analysis for significant texture predictors of PFS.

Features	Cutoff	Median PFS (months) for patients above the cutoff	Median PFS (months) for patients below/equal to the cutoff	*p*-value
Kurtosis (SSF = 2)	1.11	37.8	10.1	0.008
Kurtosis (SSF = 3)	1.20	26	13.2	0.04
*Perc*-ENTRO (SSF = 4)	5.0	18.7	10.5	0.01
*Perc*-ENTRO (SSF = 6)	5.0	18.7	10.5	0.0005

## Discussion

Our pilot study indicates that baseline and post-therapy contrast-enhanced CT-based radiomics texture features of MM have the potential to act as imaging biomarkers of outcome in terms of OS and PFS in patients treated with Nivolumab. Both kurtosis at baseline CT and percentage change in entropy without filtration, between post-treatment and baseline CT, are the best predictors of outcome and can potentially act as a predictive and response biomarker, respectively. The addition of delta-radiomics increases the available quantitative information related to the spatial and temporal intra-tumor heterogeneity (ITH), potentially reflecting tumor phenotypical changes over time that are crucial in assessing response to immunotherapy.

In the era of artificial intelligence and machine learning (ML), quantitative texture-based radiomic features extracted from medical images can provide objective information and, therefore, play an important role in decision support in cancer care pathways and patient management (28, 29). Over the past few years, CTTA has been acknowledged as a promising quantitative imaging tool allowing for measurement of the spatial ITH by analyzing the gray-level distribution of CT images (30). Previous studies explored biological correlates for CTTA, confirming an association between CT heterogeneity and a hypoxic and angiogenic tumor microenvironment (23, 30); at the same time, it is hypothesized that tumor angiogenesis (TA) may have value in predicting patient survival in different cancers (31–37).

In MM, multivariate analysis confirmed that texture variables are significant predictors of survival and suitable to build a prognostic index/composite score along with established important clinical markers, such as LDH and number of metastatic sites (4, 38, 39). We found kurtosis at fine (SSF = 2) and medium (SSF = 3) texture scales in order to best predict the outcome (OS and PFS) of patients treated with Nivolumab at baseline CT. Kurtosis is a measure of the sharpness/pointedness of distribution in the histogram of images. A higher value indicates increased tissue contrast, which could be associated with tissue vascularity (23). In our study, hypothesizing kurtosis at fine to medium texture scales may reflect contrast medium changes directly/indirectly associated with small/medium-sized blood vessels (micro-vasculature) within the tumor microenvironment.

In patients treated with immunotherapy, tumor vasculature may impact the host immune response (40). In fact, functional abnormalities of tumor blood vessels, such as architectural defects, can limit lymphocyte recruitment. A correlation between the presence of tumor-infiltrating lymphocytes (TILs) and an improved prognosis has been demonstrated in cancer patients treated with immunotherapy (40, 41). We can assume that a high value of kurtosis may reflect a higher T-cell infiltration within a lesion, resulting in a “T-cell inflamed” phenotype (“hot tumors”) associated with lower tumor blood vessel defects and a potential higher responsiveness to PD-1 inhibitor therapy (42). In a recent retrospective study, Schraag et al. have proposed that the kurtosis as an independent predictor of OS in MM patients treated with different immunotherapy regimes, corroborating our results (43). However, some important differences with our pilot study should be emphasized. Indeed, the authors of this study have involved a more heterogeneous population of MM patients (the patients enrolled were treated with CTLA4 and PD1 inhibitors) and have focused their analysis only on the largest target lesion. We believe that this choice may be limiting because it is less representative of the total tumor burden. Texture-based radiomic analysis is dependent on the variation/distribution of the pixel intensities within the ROI enclosing the lesion. Smaller lesions will have fewer pixels/distribution of gray-level intensities whereby the statistics may not be optimum. Also, the lesions <5 mm size may not be clinically relevant. This hypothesis is confirmed by RECIST and other criteria that recommend analysis of up to five target lesions. We basically employed what is normally done in routine clinical practice when assessing response to treatment in these oncological patients (e.g., RECIST 1.1 criteria dictate selecting up to five lesions per patient) to be as close and relevant to current practice. Applying different weightings to different types of lesions or only considering one lesion per patient was not explored as it is not something done routinely and there is no biological rationale “currently” to utilize this. Nevertheless, there are very interesting points to explore in the future, such as certain types (based on anatomy) of metastatic lesions could be weighed differently and could be more robust and sensitive/accurate in early prediction of responders from non-responders.

Contrary to our study that has analyzed the response to Nivolumab, Durot et al. have investigated the role of CTTA in predicting response to immunotherapy in MM patients treated with another anti-PD-1 inhibitor (Pembrolizumab). In this study, the authors reported the role of skewness (i.e., asymmetry of the histogram) as a potential predictor of outcome (31). Pembrolizumab and Nivolumab belong to the same family (anti-PD-1) and are similar (44); it has been suggested that differences observed in clinical data between these two drugs are unlikely to be drug-dependent and are likely to be due to drug-independent differences (44). We can assume that differences in our results are more likely to be due to the small patient population in both studies, which could amplify potential individual patient characteristics, as well as drug administration and imaging protocols. The potential influence of iterative algorithms and contrast administration protocols on radiomic analysis needs to be better investigated (45). In any case, we hypothesize that both kurtosis and skewness may be picking up similar image characteristics. Skewness reflects the preponderance of object brightness/darkness, which could again reflect an aspect of tumor vascularity similar to kurtosis. Therefore, both kurtosis and skewness could have a potential role in reflecting vascular and non-vascular morphological changes within the lesion. These inter-relationships and the above hypotheses need to be investigated in further studies with a larger patient population.

It is well established that greater tumor heterogeneity is an indicator of poor clinical prognosis. Furthermore, tumor-induced angiogenesis contributes to a disorganized microenvironment leading to tortuous architecture of the vasculature, eventually resulting in the formation of hypoxic voids and necrosis. Consequently, different sub-clonal cell populations within a tumor arise over time, contributing to different phenotypes (7, 40). It is important to note that phenotypic plasticity can occur due to non-genetic factors and a variation exists at multiple omics levels (46); “phenotypic” delta signatures for predicting survival could better correlate with other molecular markers over time. In the delta-radiomics analysis, the Perc-ENTRO significantly predicted both OS and PFS. Lesions with high entropy usually correlated with higher heterogeneity. In other works, entropy was correlated with the outcome of therapy (34, 37, 47).

The exhibit of a more disorganized microenvironment within the lesion may appear as increased imaging heterogeneity, i.e., higher entropy on the post-treatment scan in comparison to the baseline scan, giving rise to an increased Perc-ENTRO, a response marker indicating the worst outcome (OS and PFS) in our study. We believe that the combination of texture-based radiomic analysis and recent multi-omics approaches can help implement precision medicine and a robust decision-making tool in patients (48–51). This tool/approach is particularly relevant in a MM setting having a complex biology, inter/intra-tumor heterogeneity and is one of the most aggressive cancers (7).

Our study has some limitations. Firstly, the relatively small patient population makes our hypothesized associations speculative and exploratory in nature. Moreover, only a manual 2D analysis on cross-sectional CT images was performed. However, this was undertaken in consensus with two expert radiologists in CT oncological imaging, thereby limiting the potential bias. In any case, both approaches were found to capture heterogeneity and were good predictors of survival (52). Moreover, although our results are promising, of course a validation data set is missing and data have to be confirmed in future studies in order to validate the method.

In fact, this is a pilot study purely for exploratory purposes; the focus of this pilot/proof-of-principle study was to assess the potential of each texture parameter from the baseline and post-treatment CT scan using filtration-histogram-based technique (a technique that has undergone proper validation as evidenced from the numerous papers using this technique) to predict outcome (OS and PFS) post immunotherapy and compare to existing clinical/imaging markers. Future studies could implement a more complex ML algorithm (incorporating the significant texture features identified in this pilot study) and employ normalization/standardization approaches in a larger cohort, which could further be randomly divided into training and validation sub-cohorts. Furthermore, as the study population in this pilot/proof of principle was very small at 32 patients, the idea was not to employ complex approaches (e.g., ML and numerous radiomic features) as we do not have a dedicated training and testing dataset. Based on this exploratory study, we do propose to undertake a larger prospective study in the future where we will apply the above suggested methodologies (e.g., ML techniques), employing a comprehensive radiomic approach including the filtration-histogram-based technique in addition to higher-order statistics, shape parameters, and training and testing cohort. Another limitation of this pilot study is the lack of the assessment of intra and inter-reader agreement, as only information from segmentation from a consensus reading was performed; but we would like to point out the qualification process undertaken by the filtration-histogram based texture analysis evidenced from numerous other peer-reviewed publications.

However, we would like to point out the quantification process undertaken by the filtration-histogram-based texture analysis evidenced from numerous other peer-reviewed publications: (26, 53, 54). Specifically, the filtration step part of the texture analysis technique reduces the impact of image photon noise, thereby minimizing the impact of image acquisition variation and therefore the quantification of texture features using histogram and statistical approach reflects biologically relevant heterogeneity. The use of the filtration-histogram technique further mitigates the need for the use of larger number of higher-order statistics, which are more abstract in nature, are less reproducible, and increase false discovery rate.

## Conclusions

In conclusion, our study demonstrates the potential role of kurtosis to select MM patients with improved OS and PFS at baseline CT, as an independent predictor of outcome (“predictive-biomarker”). In delta-radiomics analysis, we found Perc-ENTRO to be a good independent predictor for both OS and PFS in MM patients treated with Nivolumab (“response-biomarker”). If this method is validated, we hypothesize that these parameters could potentially improve better patient selection and the response evaluation to immune check point inhibitors and, therefore, be used as an adjunct in decision-making and optimal patient management. On the basis of our promising preliminary results, further studies with a larger MM population treated with PD-1 inhibitors are needed to investigate the usefulness of delta-radiomics based CT texture features in a multi-omics approach.

## Data Availability Statement

The datasets presented in this article are not readily available because we are not yet able to predict whether the necessary internal approvals and permissions and patient consents can be shared, even if anonymously. Requests to access the datasets should be directed to emiliano.loi88@gmail.com.

## Ethics Statement

The studies involving human participants were reviewed and approved by IFO—Istituti Fisioterapici Ospitalieri. Written informed consent for participation was not required for this study in accordance with the national legislation and the institutional requirements.

## Author Contributions

Conceptualization: AG, MR, and AM. Data curation: EL, BG, SU, VB, and DR. Formal analysis: AG, EL, BG, VB, and FS. Investigation: AG and VF. Methodology: AG, EL, VF, and FS. Resources: MR. Supervision: AG, FC, and AM. Validation: BG. Visualization: FD and MC. Writing—original draft: AG and EL. Writing—review and editing: IF, MR, and BG. All authors contributed to the article and approved the submitted version.

## Funding

BG is affiliated to Institute of Nuclear Medicine, University College London/University College London Hospitals, which receives proportional funding through the UK National Institute of Health Research/Biomedical Research Centre funding scheme. No specific grant number is associated with the presented researcher.

## Conflict of Interest

One of the authors, BG, (who was not a data controller for this study) is the co-founder/co-inventor of TexRAD texture analysis software used in this study and a shareholder (not an employee) of Feedback Plc., a UK based company that owns, develops, and markets the TexRAD texture analysis software.

The remaining authors declare that the research was conducted in the absence of any commercial or financial relationships that could be construed as a potential conflict of interest.

## Publisher’s Note

All claims expressed in this article are solely those of the authors and do not necessarily represent those of their affiliated organizations, or those of the publisher, the editors and the reviewers. Any product that may be evaluated in this article, or claim that may be made by its manufacturer, is not guaranteed or endorsed by the publisher.
