# Supplementary material for: Exploring CT Texture Parameters as Predictive and Response Imaging Biomarkers of Survival in Patients With Metastatic Melanoma Treated With PD-1 Inhibitor Nivolumab: A Pilot Study Using a Delta-Radiomics Approach
Source: Front Oncol. 2021 Oct 7;11:704607. doi: 10.3389/fonc.2021.704607 (PMC8529867; doi:10.3389/fonc.2021.704607)
Supplement: Supplementary file 1 [file Table_1.docx]

**Table 1S.** CT parameters used in this study

| Brilliance CT, Philips Healthcare | 128 MDCT scanner |
| --- | --- |
| detector width (mm) | 64 x 0.625 (128) |
| tube voltage | 100 kVp |
| tube current modulation thecnique (ATCM) | 200 mAs |
| pitch | 0.891 |
| rotation time | 0.4 s |
| field of view (FOV) | 350 mm |
| slice thickness | 2.5 mm |
| slice increment | 1.25 mm |
| matrix | 512 X 512 |
| pixel spacing | 0.98 mm X 0.98 mm |
