# Supplementary material for: Exploring CT Texture Parameters as Predictive and Response Imaging Biomarkers of Survival in Patients With Metastatic Melanoma Treated With PD-1 Inhibitor Nivolumab: A Pilot Study Using a Delta-Radiomics Approach
Source: Front Oncol. 2021 Oct 7;11:704607. doi: 10.3389/fonc.2021.704607 (PMC8529867; doi:10.3389/fonc.2021.704607)
Supplement: Supplementary file 2 [file Table_2.docx]

| **Filter (Spatial scale filter - SSF in mm)** | **Texture-parameter** |
| --- | --- |
| 0 (without-filtration) | Mean intensity |
|  | Standard-deviation |
|  | Entropy |
|  | Mean of positive pixels |
|  | Skewness |
|  | Kurtosis |
| 2 (fine-scale) | Mean intensity |
|  | Standard-deviation |
|  | Entropy |
|  | Mean of positive pixels |
|  | Skewness |
|  | Kurtosis |
| 3 (medium-scale) | Mean intensity |
|  | Standard-deviation |
|  | Entropy |
|  | Mean of positive pixels |
|  | Skewness |
|  | Kurtosis |
| 4 (medium-scale) | Mean intensity |
|  | Standard-deviation |
|  | Entropy |
|  | Mean of positive pixels |
|  | Skewness |
|  | Kurtosis |
| 5 (medium-scale) | Mean intensity |
|  | Standard-deviation |
|  | Entropy |
|  | Mean of positive pixels |
|  | Skewness |
|  | Kurtosis |
| 6 (coarse-scale) | Mean intensity |
|  | Standard-deviation |
|  | Entropy |
|  | Mean of positive pixels |
|  | Skewness |
|  | Kurtosis |

Table 2S

List of the TA features extracted from the CT images; for each Spatial Scale Filter the first order statistic features were extracted, including mean (average grey-level intensity values), standard deviation (SD; degree of variation of pixel values), entropy (irregularity of grey-level distribution), mean of positive pixels (MPP; pixel with values greater than 0), skewness (asymmetry of the histogram) and kurtosis (reflects pointedness/sharpness of the histogram distribution)
